# Estrogen associations with human pregnancy related increases in cytochrome P450 3A activity

**DOI:** 10.3389/fphar.2025.1702419

**Published:** 2025-11-26

**Authors:** Muluneh M. Fashe, Jonghwa Lee, Joseph T. Grieco, John K. Fallon, Ian R. Mulrenin, Megan N. Gower, Klarissa D. Jackson, Kim A. Boggess, Craig R. Lee

**Affiliations:** 1 Division of Pharmacotherapy and Experimental Therapeutics, UNC Eshelman School of Pharmacy, University of North Carolina at Chapel Hill, Chapel Hill, NC, United States; 2 Division of Pharmacoengineering and Molecular Pharmaceutics and Center for Nanotechnology in Drug Delivery, UNC Eshelman School of Pharmacy, University of North Carolina at Chapel Hill, Chapel Hill, NC, United States; 3 Department of Obstetrics and Gynecology, School of Medicine, University of North Carolina at Chapel Hill, Chapel Hill, NC, United States

**Keywords:** pregnancy, CYP3A4, hepatic metabolism, estrone, estradiol, 4β-hydroxycholesterol

## Abstract

**Introduction:**

Increased CYP3A-mediated drug clearance during pregnancy can lead to subtherapeutic dosing of CYP3A substrates. Pregnancy-related hormones (PRHs) increase CYP3A4 expression and activity in cultured human hepatocytes. However, the factors in maternal circulation that regulate pregnancy-mediated changes in CYP3A activity remain unclear.

**Methods:**

This study investigated the association between maternal plasma concentrations of key steroidal PRHs and biomarkers of CYP3A activity in human pregnancy, and the impact of individual PRHs on CYP3A4 expression in primary human hepatocytes. Concentrations of estrone (E1), estradiol (E2), progesterone (P4), and cortisol (CRT), and 4*β*-hydroxycholesterol (4*β*-OH-CHO) and the 4*β*-OH-CHO:CHO ratio (endogenous biomarkers of CYP3A activity), were quantified in human plasma across a spectrum of pregnancy states: healthy nonpregnant controls (n = 4), healthy pregnant volunteers (n = 6), and pregnant patients diagnosed with preeclampsia (n = 8).

**Results:**

Plasma 4*β*-OH-CHO concentrations (median [25%–75%]) were higher in healthy pregnant (141 [115, 165] ng/mL) and preeclampsia patients (129 [90.5, 191] ng/mL) compared to nonpregnant controls (69.8 [45.8, 82.5] ng/mL). In healthy pregnant and preeclampsia patients, plasma E1 (*r = 0.687, p = 0.007*) and E2 (*r = 0.551, p = 0.041*) concentrations positively correlated with plasma 4*β*-OH-CHO concentrations. Conversely, no association between P4 (*r = 0.068, p = 0.817*) or CRT (*r = -0.115, p = 0.696*) concentrations and 4*β*-OH-CHO were observed. Cultured human female primary hepatocytes were exogenously exposed *in vitro* to PRHs and absolute CYP protein concentrations were quantified. Consistent with the human plasma sample associations, E1 and E2 induced CYP3A4 mRNA and total CYP3A protein concentrations in a concentration-dependent manner.

**Discussion:**

Altogether, these data suggest that increased concentrations of E1 and E2 contribute, at least in part, to increased hepatic CYP3A expression and activity during pregnancy in humans.

## Introduction

Approximately 90% of pregnant people take at least one medication during gestation (Mitchell et al., 2011; Werler et al., 2023; Hwang et al., 2024). Despite the widespread use of medications in this patient population, very few drugs are approved for use during pregnancy and pregnant individuals rarely participate in clinical drug trials. It is estimated that only 10% of drugs have pregnancy data from human studies or have pregnancy specific dosing recommendations, resulting in a substantial gap in knowledge regarding standardized drug dosing, efficacy, and safety information (Byrne et al., 2020).

Clinical studies have demonstrated that pregnancy alters the pharmacokinetics (PK) of various drugs that are cleared by certain hepatic drug-metabolizing enzymes (DMEs) such as cytochrome P450s (CYPs) (Pariente et al., 2016). A notable example is the pregnancy-related increase in CYP3A metabolic activity (Hebert et al., 2008; Mlugu et al., 2022). CYP3A is responsible for the clearance of >50% of drugs, including various commonly prescribed medications during pregnancy such as buprenorphine, nifedipine, glyburide, dexamethasone and clindamycin, and maternal oral clearance of various CYP3A substrates has been reported to increase during gestation in humans (Anderson, 2005; Mitchell et al., 2011; Mulrenin et al., 2021). Compared to nonpregnant controls, multiple studies have reported that plasma concentrations of 4*β*-hydroxycholesterol (4β-OH-CHO, a CYP3A metabolite of cholesterol) and the ratio of 4β-OH-CHO to cholesterol (4β-OH-CHO:CHO), known biomarkers of CYP3A activity *in vivo*, are increased during pregnancy (Kim et al., 2018; Mlugu et al., 2022). Similarly, increased CYP3A-mediated metabolite formation from the CYP3A substrate drugs midazolam and buprenorphine during pregnancy has been reported (Hebert et al., 2008; Hollabaugh et al., 2019; Zhang et al., 2020), collectively demonstrating evidence of increased CYP3A-mediated metabolism of multiple substrates during gestation. Furthermore, it has been observed that pregnant patients diagnosed with preeclampsia have higher plasma 4β-OH-CHO concentrations relative to normotensive pregnancies (Moon et al., 2014); however, preeclampsia-associated changes in CYP3A activity require further study. The observed increase in CYP3A activity during pregnancy has been linked to induction of CYP3A4 expression in maternal liver by pregnancy related hormones (PRHs) (Jeong, 2010; Isoherranen and Thummel, 2013). However, the key factors in maternal circulation that regulate pregnancy-mediated changes in CYP3A activity in healthy pregnancies and those complicated by pregnancy-related conditions such as preeclampsia (that is frequently treated with the CYP3A substrate nifedipine) necessitate further study.

The hypothesis that PRHs trigger the transcriptional induction of CYP3As has attracted considerable research interest and is supported by the observation that steroidal PRHs such as cortisol (CRT), estrone (E1), estradiol (E2), and progesterone (P4) circulate at very high concentrations in pregnant individuals and can activate their natural receptors or the xenosensors pregnane X receptor (PXR) and constitutive androstane receptor (CAR) (Kawamoto et al., 2000; Xue et al., 2007; Newbern and Freemark, 2011). To test the hypothesis, several models, such as hepatocyte cultures *in vitro* and experimental animals *in vivo*, have been developed. In studies with human primary hepatocytes, exposure to PRHs significantly increased *CYP3A4* mRNA levels, CYP3A4 protein expression, and CYP3A activity towards the probe substrate midazolam, as well as the drug substrates nifedipine and buprenorphine (Choi et al., 2013; Papageorgiou et al., 2013; Khatri et al., 2021; Fashe et al., 2023). In wild type mice, pregnancy increased CYP3A activity due to increased expression of hepatic female-predominant murine Cyp3a isoforms despite a substantially reduced expression of *Cyp3a11* mRNA, a major murine Cyp3a (Shuster et al., 2013; Sriwattanapong et al., 2017). Similarly, in transgenic mice carrying a CYP3A4-promoter harboring luciferase gene reporter, pregnancy activated the CYP3A4 promoter (Zhang et al., 2008; Sriwattanapong et al., 2017). Together, these data suggest that pregnancy increases hepatic CYP3A activity by transcriptional induction of CYP3A4 expression in the liver. However, the association between elevated secretion of steroidal PRHs and increased CYP3A activity during human pregnancy has remained unclear.

The primary objectives of the current study were to (1) evaluate the association between plasma concentrations of key steroidal PRHs (E1, E2, P4, or CRT) and endogenous biomarkers of CYP3A activity (4β-OH-CHO, 4β-OH-CHO:CHO ratio) across a spectrum of pregnancy states in humans, and (2) experimentally evaluate the impact of individual PRHs on CYP3A4 and total CYP3A absolute protein concentrations in primary human hepatocytes. To achieve these goals, we quantified 4β-OH-CHO, the 4β-OH-CHO:CHO ratio, and steroidal PRHs (E1, E2, P4, and CRT) in plasma obtained from healthy nonpregnant, healthy pregnant, and pregnant patients diagnosed with preeclampsia, and quantified the effects of PRHs on the absolute protein concentration of CYP3A4 (and other key CYP isoforms) in sandwich cultured human hepatocytes (SCHH).

## Materials and methods

### Study participants and design

This study was carried out in plasma samples collected from participants enrolled in study NCT03419364. The study design and participant characteristics have been previously described (Fashe et al., 2024). Briefly, the study included an ancillary single-dose PK study of nicotinamide in healthy pregnant and nonpregnant volunteers (n = 6/group) and an interventional open-label single-arm study of nicotinamide in early-onset pre-eclampsia patients (n = 8 trial participants with a morning pre-dose sample collected). For the healthy nonpregnant group, inclusion criteria were age 18–45 years, a negative pregnancy test, and no chronic medical conditions; in this group, two participants from the original study were excluded from the current analysis due to reported use of bupropion, a CYP substrate. Additional criteria in the healthy pregnancy group included gestational age between 24 and 33 weeks, a singleton pregnancy with no known fetal anomalies, and no chronic or pregnancy-associated medical condition requiring treatment. In both groups, current smokers and individuals with diabetes, hypertension, or renal disease (creatinine ≥1.5 mg/dL) were excluded. Inclusion and exclusion criteria for the pregnant patients diagnosed with early-onset preeclampsia (gestational age 24–33 weeks) are summarized at ClinicalTrials.Gov (Boggess, 2022; Fashe et al., 2024). Briefly, all patients exhibited preeclampsia with a severe feature including severe gestational hypertension (systolic blood pressure ≥160 and/or diastolic ≥105 mmHg) or hypertension (systolic blood pressure ≥140 and/or diastolic ≥90 mmHg) with certain signs or symptoms of end-organ dysfunction (e.g., proteinuria, doubling of serum creatinine from baseline, or visual changes); patients with liver dysfunction, thromobocytopenia, HELLP syndrome, or other more severe features were excluded. The preeclampsia patients could receive antihypertensive agents per their clinical care.

The ancillary PK study and interventional study was conducted in the UNC Clinical Translational Research Center and UNC Medical Center, respectively. Participants were not required to fast prior to the study visit. Blood samples used in the current analysis were collected in EDTA tubes (Fisher Scientific) in the morning before nicotinamide administration (t = 0). Plasma was separated by centrifugation and stored at −80 °C. The UNC-Chapel Hill Institutional Review Board approved the study protocol, and all the procedures were conducted according to the Declaration of Helsinki.

### Reagents

Reagents were obtained from Thermo Fisher Scientific (Waltham, MA) unless otherwise indicated.

### Quantification of 4β-OH-CHO and CHO

4β-OH-CHO and free CHO were quantified using liquid chromatography–tandem mass spectrometry (LC-MS/MS), as previously described with minor modification (Lee et al., 2023; Fashe et al., 2025). Briefly, plasma samples were diluted in Dulbecco’s phosphate-buffered saline (PBS) buffer (pH, 7.4) for the quantification of free CHO (1:250). Calibration samples (50 ng/mL – 4,000 ng/mL for free CHO and 1.5 ng/mL – 300 ng/mL for 4β-OH-CHO) were prepared in isopropanol. A mixture of 50 µL study samples (calibration samples, diluted plasma for free CHO or undiluted plasma for 4β-OH-CHO) and 25 µL butylated hydroxytoluene (1 mg/mL) were prepared in glass tubes and spiked with d_7_-CHO (1,000 ng/mL) or d_7_-4β-OH-CHO (100 ng/mL) analytical standards for free CHO or 4β-OH-CHO assay, respectively (Toronto Research Chemicals, Canada). 4β-OH-CHO samples were hydrolyzed by adding 250 µL potassium hydroxide in ethanol (1 M) at 37 °C for 1 h. To both free CHO and 4β-OH-CHO samples, 500 µL hexane and 140 µL water were added and centrifuged at 4,000 rpm for 5 min at 4 °C. 300 μL of the supernatant was transferred to fresh Eppendorf tubes, evaporated to dryness, and resuspended in 170 µL of derivatization mixture containing 2-methyl-6-nitrobenzoic anhydride (1,224.5 mg), 4-dimethylaminopyridine (373.3 mg), picolinic acid (995.5 mg), and triethylamine (2.5 mL), and pyridine (1.87 mL). The mixture was incubated at room temperature for 30 min followed by extraction with 500 µL hexane and centrifugation at 4,000 rpm for 5 min at 4 °C. Finally, 300 µL of the supernatant was transferred to a fresh tube and evaporated to dryness in N_2_ gas and reconstituted in 200 µL acetonitrile for quantification by LC-MS. Chromatographic separation and subsequent quantification were achieved by injecting the 10 µL sample to a Waters Acquity ultraperformance liquid chromatography (UPLC) system (Milford, MA) equipped with a Sample Manager (part number 186015005), Binary Solvent Manager (part number 186015001), and Waters Acquity UPLC BEH C18 column (1.7 μm, 100 × 2.1 mm) coupled to a Thermo Finnigan TSQ Quantum Ultra triple quadrupole mass spectrometer system (Thermofisher, Waltham, MA). 4β-OH-CHO and free CHO LC-MS/MS data were acquired and analyzed on Xcalibur™ Software version 3.0.63 (Thermofisher Scientific, Waltham, MA). 4β-OH-CHO concentrations are presented in ng/mL and free CHO concentrations are presented in mg/dL. The ratio of 4β-OH-CHO to free CHO was calculated in each participant by dividing the plasma 4β-OH-CHO concentration by the free CHO concentration and multiplying by 10^4^.

### Quantification of estrone, estradiol, progesterone, and cortisol

Plasma concentrations of E1, E2, P4, and CRT were quantified using commercially available enzyme-linked immunosorbent assay (ELISA) kits according to the manufacturer instructions. Briefly, E1 was quantified using an Estrone ELISA kit (ALPCO, Salem, NH); plasma aliquots were diluted in PBS (1:4 for nonpregnant and 1:49 for pregnant), and then 50 µL of the diluted samples were used to quantify E1 per the manufacturer’s guidelines. E2, P4, and CRT were quantified using their respective ELISA kits (Cayman, Ann Arbor, MI). For E2 quantification, plasma was diluted in pure water (6:19 for non-pregnant and pregnant 3:47 for pregnant), and then 50 µL of the diluted plasma was mixed with four volumes of methanol, incubated at room temperature for 10 min, and centrifuged at 2000 x g for 10 min. The supernatant was collected, evaporated to dryness, reconstituted in ELISA buffer, and stored in −80 °C until the quantification assay. For the P4 assay, plasma samples were diluted in pure water (2:23 for non-pregnant and 1:24 for pregnant) prior to extraction with 3% isopropanol in hexane (v/v). Then, 50 µL of the diluted plasma was mixed with four volumes of 3% isopropanol in hexane and the organic phase was pooled in a clean tube, evaporated to dryness, reconstituted in ELISA buffer, and stored in −80 °C until use. For the quantification of CRT, plasma was diluted in pure water (1:24 for both non-pregnant and pregnant, pH 2) prior to extraction with ethyl acetate. The organic phase was pooled in fresh tubes, evaporated to dryness, reconstituted in ELISA buffer, and stored in −80 °C until use. All assays were performed in triplicate and plasma sample concentrations were quantified using a standard curve per manufacturer’s guidelines. We did not measure estriol (E3) and placental growth hormone (pGH) concentrations due to an insufficient amount of plasma and lack of a reliable analytical method, respectively.

### Sandwich-cultured human hepatocytes and quantitative targeted absolute proteomics (QTAP)

Cryopreserved human hepatocytes derived from five female donors of reproductive age (18–49 years) were obtained from Life Technologies Corporation (Carlsbad, CA) (Hu8339, Hu8373, Hu8375, Hu 1970) or BioIVT (Durham, NC) (YNM). Hepatocyte donor characteristics are summarized in Supplementary Table S1. Methods for SCHH culture, PRH treatment, membrane protein fractionation, and quantification of the absolute protein concentrations have been previously described (Fashe et al., 2022; Fashe et al., 2023). Briefly, on day 1, hepatocytes were seeded in QualGro™ seeding medium (BioIVT) at a density of 250,000 cells/well (QTAP) or 150,000 cells/well (mRNA) in collagen coated 24- or 48-well plates, respectively (Corning, NY) overnight. On day 2, the seeding medium was aspirated, and SCHHs were maintained in QualGro™ culture medium (BioIVT) supplemented with 0.25 mg/mL Corning Matrigel® Matrix (Corning) for 24 h. On days 3–5 (over 72 h), SCHHs were exogenously exposed to vehicle control (0.1% DMSO) or a cocktail of PRHs (E1, E2, E3, P4, CRT, and pGH) targeting the average trimester 2 (T2), average trimester 3 (T3), and upper range of T3 (T3-90th %) plasma concentration of each hormone in maternal circulation. The PRH target and treatment concentrations, and the rationale for treatment concentration selection, have been presented in detail previously (Fashe et al., 2022) and are summarized in Table 1. To study the concentration-dependent effect of each individual PRH, secondary experiments were undertaken with three hepatocyte donors (Hu8373, Hu8375, and Hu1970) where SCHH were exposed to the average T3 concentration of each individual PRH contained in the cocktail or a 10-fold higher than T3 (10x-T3) supraphysiological concentration of each individual PRH; the target and treatment T3 and 10-T3 concentrations of each PRH are summarized in Table 1. In all experiments, the culture medium was replenished twice a day (at 9 a.m. and 5 p.m.). On day 6, cytosolic and membrane proteins were isolated using a previously reported differential detergent fractionation method (Qasem et al., 2021; Fashe et al., 2022). The protein fractions were stored at −80 °C until use.

**TABLE 1:** Target and treatment concentrations of pregnancy related hormones (PRH) in sandwich-cultured human primary hepatocytes (SCHH).

PRH	Average target concentration				Treatment concentration			
	T2	T3	T3-90%	10x-T3*	T2	T3	T3-90%	10x-T3*
E1 (nM)	18	42	70	420	125	250	450	2,500
E2 (nM)	37	80	121	800	225	500	750	5,000
E3 (nM)	18	33	54	330	125	250	450	2,500
P4 (nM)	164	424	636	4,240	1,000	2,500	3,750	25,000
CRT (nM)	800	800	1,300	8,000	800	800	1,300	8,000
pGH (nM)	0.35	1.34	3.13	13.4	0.35	1.34	3.13	13.4

As previously reported in detail (Fashe et al., 2022), the listed treatment concentrations (nM) are the concentrations of each PRH exogenously administered in combination as a cocktail to the SCHH medium in the experimental model. Based on previously estimated elimination half-life estimates of each PRH in cultured human hepatocytes, these are the PRH treatment concentrations needed to achieve the desired average concentration of each PRH in SCHH over the treatment period that target the average trimester 2 (T2), average trimester 3 (T3), and upper range of T3 (T3-90%) circulating concentrations in human pregnancy. Because E1, E2, E3, and P4 are efficiently metabolized in human hepatocytes (estimated half-life 1.5–2 h), the treatment concentrations need to be increased to compensate for the metabolic loss of estrogens and P4. *10x-T3 represents a supraphysiological concentration of each individual PRH that is 10-fold higher than the average T3 concentration; the 10x-T3 treatment was only used in the individual hormone experiment.

The absolute protein concentrations of the 13 major CYP enzymes (CYP1A2, CYP2A6, CYP2B6, CYP2C8, CYP2C9, CYP2C19, CYP2D6, CYP2E1, CYP2J2, CYP3A4, CYP3A5, CYP3A7, and CYP4F2) were quantified in SCHH membrane fractions (20 µg) using a previously described quantitative targeted absolute proteomics (QTAP) nanoLC-MS/MS method (Fallon et al., 2013; Khatri et al., 2019; Fashe et al., 2023), with stable isotope labelled peptides being used as internal quantifiers (Supplementary Table S2). Data were visualized and quantified using Skyline 23.1 (University of Washington, WA) (Pino et al., 2020). The total CYP3A absolute protein concentration in each sample was quantified by calculating the sum of CYP3A4, CYP3A5, and CYP3A7 concentrations.

Total RNA was isolated from SCHH lysates using RNeasy Miniprep Kit (Qiagen, Valencia, CA) and reverse transcribed using a High-Capacity cDNA Reverse Transcription Kit (Applied Biosystems, Foster City, CA) per manufacturers’ guidelines. Quantitation of mRNA levels was performed on Applied Biosystems QuantStudio™ 6 Flex System using Taqman® gene expression assays for human *CYP3A4* (Hs00604506_m1) and *GAPDH* (Hs02758991_g1). CYP mRNA levels were normalized to *GAPDH* and vehicle control using the 2^−ΔΔCt^ method, as described (Khatri et al., 2021).

### Data analysis

Data are presented as mean ± standard deviation (SD) or median (interquartile range, 25%, 75%), and were log-transformed prior to analysis, unless otherwise indicated. Plasma concentrations of PRHs, free CHO, 4β-OH-CHO, and 4β-OH-CHO:CHO were compared between the healthy nonpregnant and healthy pregnant groups, as well as between the healthy pregnant and preeclampsia pregnant groups, using Student’s t-tests. To estimate the fold-difference in concentrations across groups for each biomarker, individual values from the healthy pregnant group were divided by the median value of the corresponding metabolite in the nonpregnant control group (similar fold-difference calculations were also completed for the preeclampsia group relative to the healthy pregnant group). Pearson correlation analysis on log-transformed data was conducted to assess the relationship between plasma concentrations of E1, E2, P4, and CRT with 4β-OH-CHO concentration and the 4β-OH-CHO:CHO ratio. Analysis of the CYP proteomics and mRNA data in SCHH across hepatocyte donors was conducted as previously described (Fashe et al., 2022). Briefly, the fold-difference in absolute protein concentration of each CYP protein following PRH exposure, relative to vehicle control, was calculated within each donor (n = 3–4 replicates per donor). Then, the average fold-difference for each treatment group within each donor was carried forward as a single data point to estimate the impact of PRH across hepatocyte donors. Comparisons across PRH groups for each CYP protein were completed using a one-way ANOVA with a *post hoc* Fisher’s LSD test to assess the effect of each PRH concentration *versus* vehicle. In each analysis, P < 0.05 was considered statistically significant. Data analysis was completed using GraphPad Prism 10.4 (San Diego, CA), SAS JMP Pro 17.0 (Cary, NC), and Microsoft Excel (Seattle, WA).

## Results

### Characteristics of study population

Characteristics of the study population are presented in Supplementary Table S3. The median age in the nonpregnant group was lower than the healthy pregnant group (24.5 vs. 33.5 years, *p < 0.001*), while the median maternal age in the healthy pregnant and preeclampsia groups was similar (33.5 vs. 29.0 years, *p = 0.145*). The median body mass index also did not significantly differ across the healthy pregnant and preeclampsia groups (26.4 vs. 31.2 kg/m2, *p = 0.197*). The median gestational age in the healthy pregnant group was lower than the preeclampsia patients (27.1 vs. 31.4 weeks, *p < 0.001*).

### Impact of pregnancy and preeclampsia on plasma concentrations of key steroidal PRHs

**FIGURE 1:**
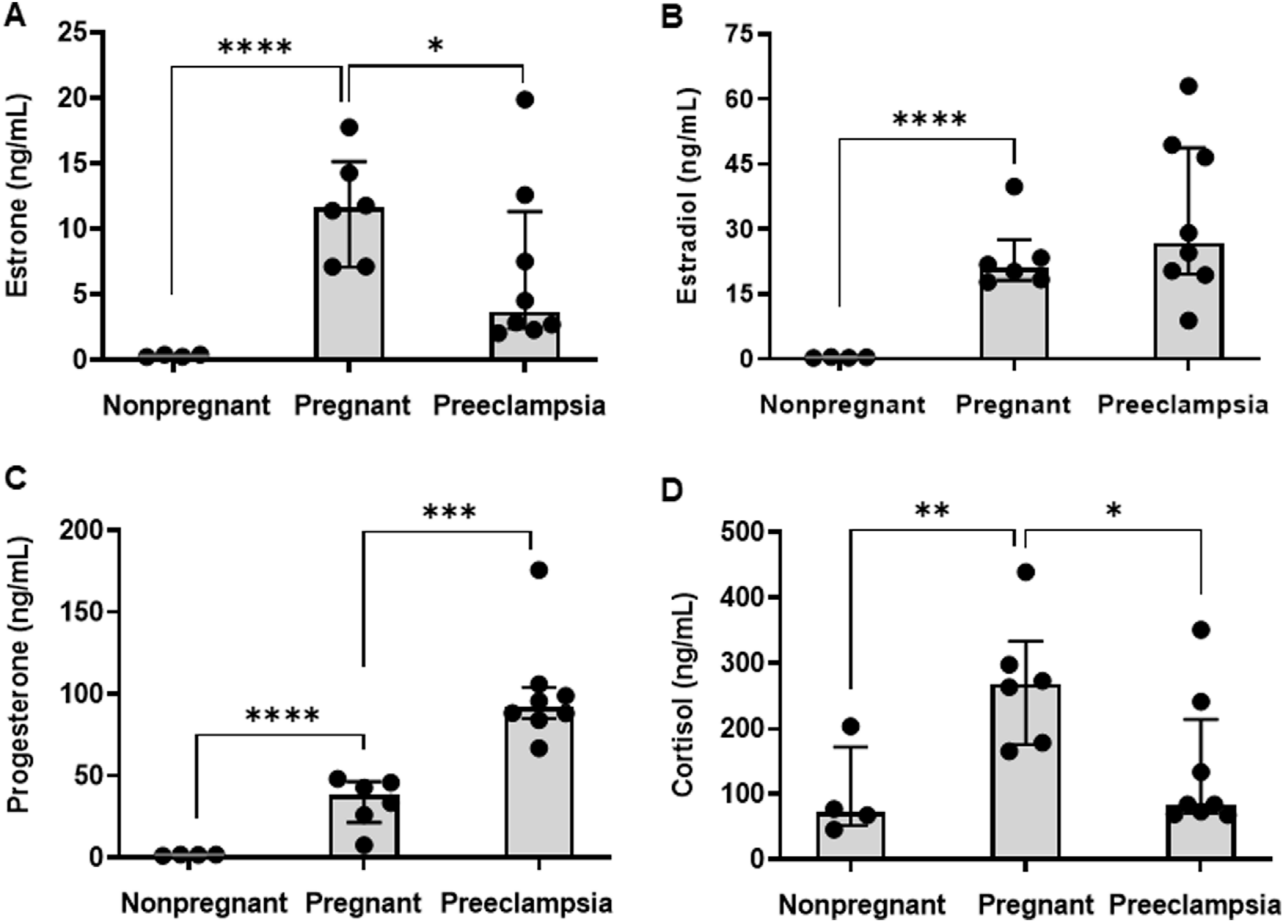
Plasma concentrations of steroidal pregnancy related hormones. The plasma concentrations of **(A)** estrone (E1), **(B)** estradiol (E2), **(C)** progesterone (P4), and **(D)** cortisol (CRT) were compared across nonpregnant (n = 4), healthy pregnant (n = 6), and preeclamptic individuals (n = 8). Data are presented as median (interquartile range). Comparisons between the pregnant and nonpregnant, and the pregnant and preeclampsia, groups were performed using a Student’s t-test on log-transformed data. *p < 0.05, **p < 0.01, ***p < 0.001, ****p < 0.0001.

Concentrations of E1, E2, P4, and CRT in maternal plasma were quantified in healthy nonpregnant volunteers, healthy pregnant volunteers, and pregnant patients diagnosed with preeclampsia. As expected, plasma concentrations of each hormone were significantly higher in pregnant volunteers compared to nonpregnant controls (Table 2; Figure 1). The median (25%, 75%) concentrations of E1 (Figure 1A), E2 (Figure 1B), P4 (Figure 1C), and CRT (Figure 1D) were 35.2 (24.8, 41.4)-fold, 58.3 (52.1, 63.7)-fold, 20.8 (15.3, 24.6)-fold, and 3.7 (2.8, 4.0)-fold higher, respectively, in pregnant volunteers relative to nonpregnant controls.

**TABLE 2:** Plasma concentrations of 4β-hydroxycholesterol (4β-OH-CHO) and steroidal pregnancy related hormones in nonpregnant, healthy pregnant, preeclampsia patients.

Metabolite	Healthy nonpregnant	Healthy pregnant	Pregnant with preeclampsia	P-value^a^ pregnant vs. nonpregnant	P-value^a^ preeclampsia vs. pregnant
N	4	6	8		
E1 (ng/mL)	0.33 (0.23, 0.41)	11.6 (7.1, 15.2)	3.69 (2.40, 11.3)	<0.0001	0.049
E2 (ng/mL)	0.36 (0.27, 0.43)	21.0 (18.1, 27.4)	26.8 (19.6, 48.7)	<0.0001	0.472
P4 (ng/mL)	1.83 (1.29, 1.95)	38.2 (21.6 46.5)	92.1 (85.2, 104)	<0.0001	0.001
CRT (ng/mL)	72.2 (51.2, 172)	268 (175, 333)	84.0 (70.2, 214.2)	0.007	0.015
4β-OH-CHO (ng/mL)	69.8 (45.8, 82.5)	141 (115, 165)	129 (90.5, 191)	0.003	0.934
Free CHO (mg/dL)	44.7 (33.5, 54.6)	63.2 (59.1, 65.9)	74.7 (59.7, 87.9)	0.009	0.353
4β-OH-CHO/CHO^a^10^4^	1.48 (1.35, 1.60)	2.16 (1.79, 2.81)	2.16 (1.73, 2.89)	0.019	0.892

Data are expressed as median (25%, 75%).

4β-OH-CHO, 4β-hydroxycholesterol; CHO, cholesterol; CRT, cortisol; E1, estrone; E2, estradiol; P4, progesterone.

^a^ Comparisons across the designated groups were analyzed by Student’s t-test on log transformed data.

Differences in plasma hormone concentrations between the preeclampsia and healthy pregnancy groups were smaller in magnitude than the differences observed between the healthy pregnant and non-pregnant groups and varied by hormone (Table 2; Figure 1). Compared to healthy pregnancy, pregnant patients diagnosed with preeclampsia exhibited 0.32 (0.22, 0.76)-fold and 0.31 (0.27, 0.60)-fold lower plasma concentrations of E1 (Figure 1A) and CRT (Figure 1D), respectively. In contrast, preeclampsia patients had 2.4 (2.3, 2.6)-fold higher P4 concentrations (Figure 1C) and no difference in E2 concentrations (Figure 1B) compared to healthy pregnant volunteers.

### Impact of pregnancy and preeclampsia on endogenous biomarkers of CYP3A activity

**FIGURE 2:**
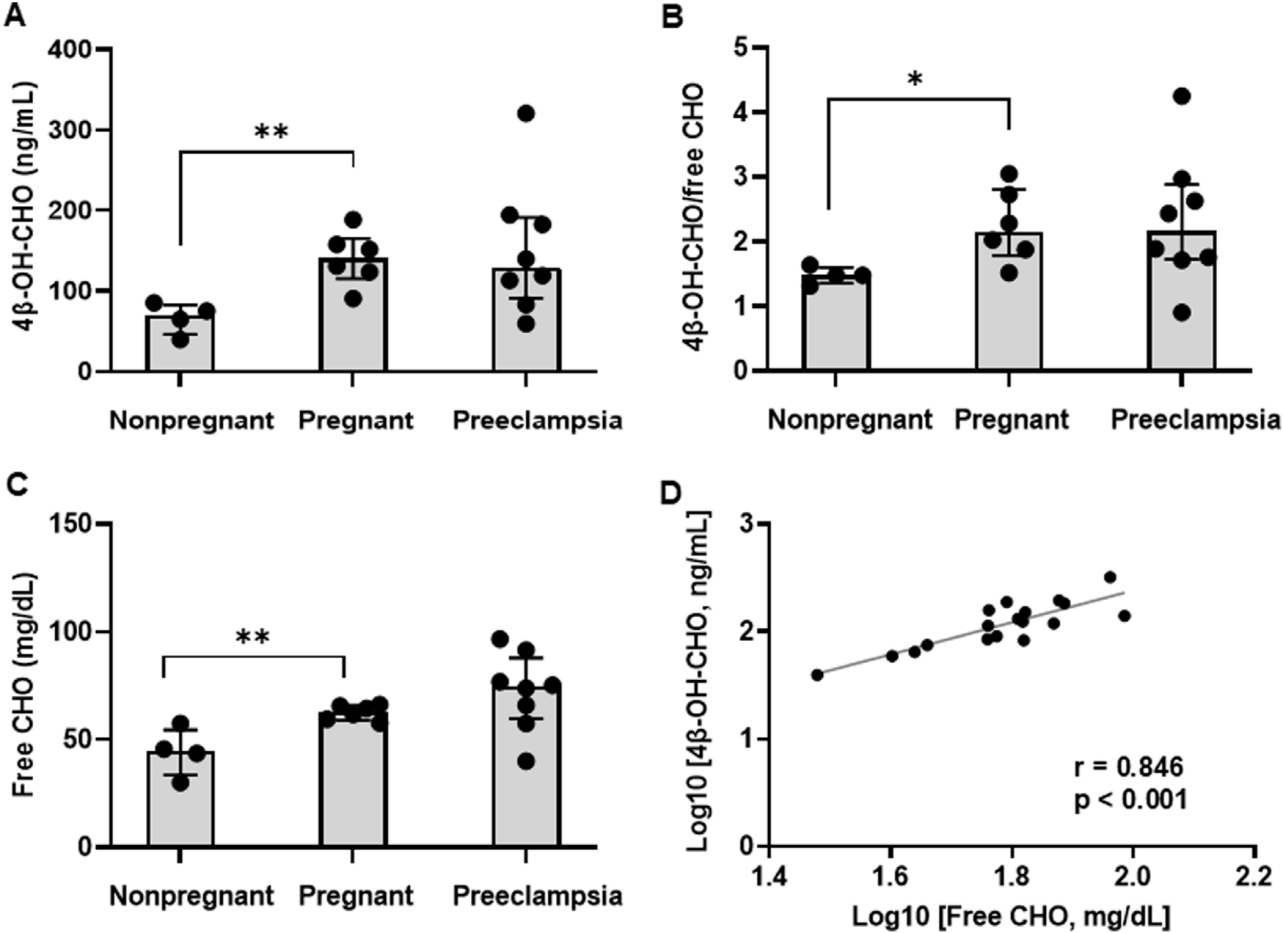
Plasma 4β-hydroxycholesterol biomarkers of CYP3A activity. Plasma **(A)** 4*β*-hydroxycholesterol (4β-OH-CHO) concentrations, and **(B)** ratio of 4β-OH-CHO to free cholesterol (CHO), and **(C)** free CHO concentrations were compared across nonpregnant (n = 4), healthy pregnant (n = 6), and preeclamptic individuals (n = 8). Data are presented as median (interquartile range). Comparisons were performed between the pregnant and nonpregnant, and the pregnant and preeclampsia, groups were performed using a Student’s t-test on log-transformed data. *p < 0.05, **p < 0.01. **(D)** Correlation between free CHO and 4β-OH-CHO (log-transformed) in nonpregnant, healthy pregnant, and preeclampsia individuals. The Pearson correlation coefficient (*r*) and *p* value are presented.

To evaluate CYP3A metabolism across a spectrum of pregnancy states in humans, plasma 4β-OH-CHO concentrations and the plasma 4β-OH-CHO to CHO ratio (endogenous biomarkers of CYP3A activity), were quantified in healthy nonpregnant volunteers, healthy pregnant volunteers, and pregnant patients diagnosed with preeclampsia (Table 2). Plasma 4β-OH-CHO concentrations were 2.02 (1.80, 2.24)-fold higher in healthy pregnant volunteers (141 [115, 165] ng/mL) compared to healthy nonpregnant controls (69.8 [45.8, 82.5] ng/mL) (Figure 2A, *p = 0.003*). Similarly, the plasma 4β-OH-CHO:CHO ratio was 1.46 (1.29, 1.77)-fold higher in the healthy pregnant group (2.16 [1.79, 2.81]) compared to nonpregnant controls (1.48 [1.35, 1.60) (Figure 2B, *p = 0.019*). In pregnant patients diagnosed with preeclampsia, 4β-OH-CHO concentrations (129 (90.5, 191) ng/mL) and 4β-OH-CHO:CHO ratios (2.16 [1.73, 2.89]) did not differ compared to healthy pregnant volunteers (*p = 0.934* and *p = 0.892*, respectively).

Free CHO concentrations in plasma were 1.41 (1.35, 1.46)-fold higher in the healthy pregnant group compared to nonpregnant volunteers (*p = 0.009*), but did not differ between the preeclampsia and healthy pregnant groups (*p = 0.353*) (Table 2; Figure 2C). As expected, plasma concentrations of 4β-OH-CHO and its precursor free CHO were positively correlated (Figure 2D, *r = 0.846*, *p < 0.001*).

### Association between steroidal PRHs and endogenous biomarkers of CYP3A activity in pregnant individuals

**FIGURE 3:**
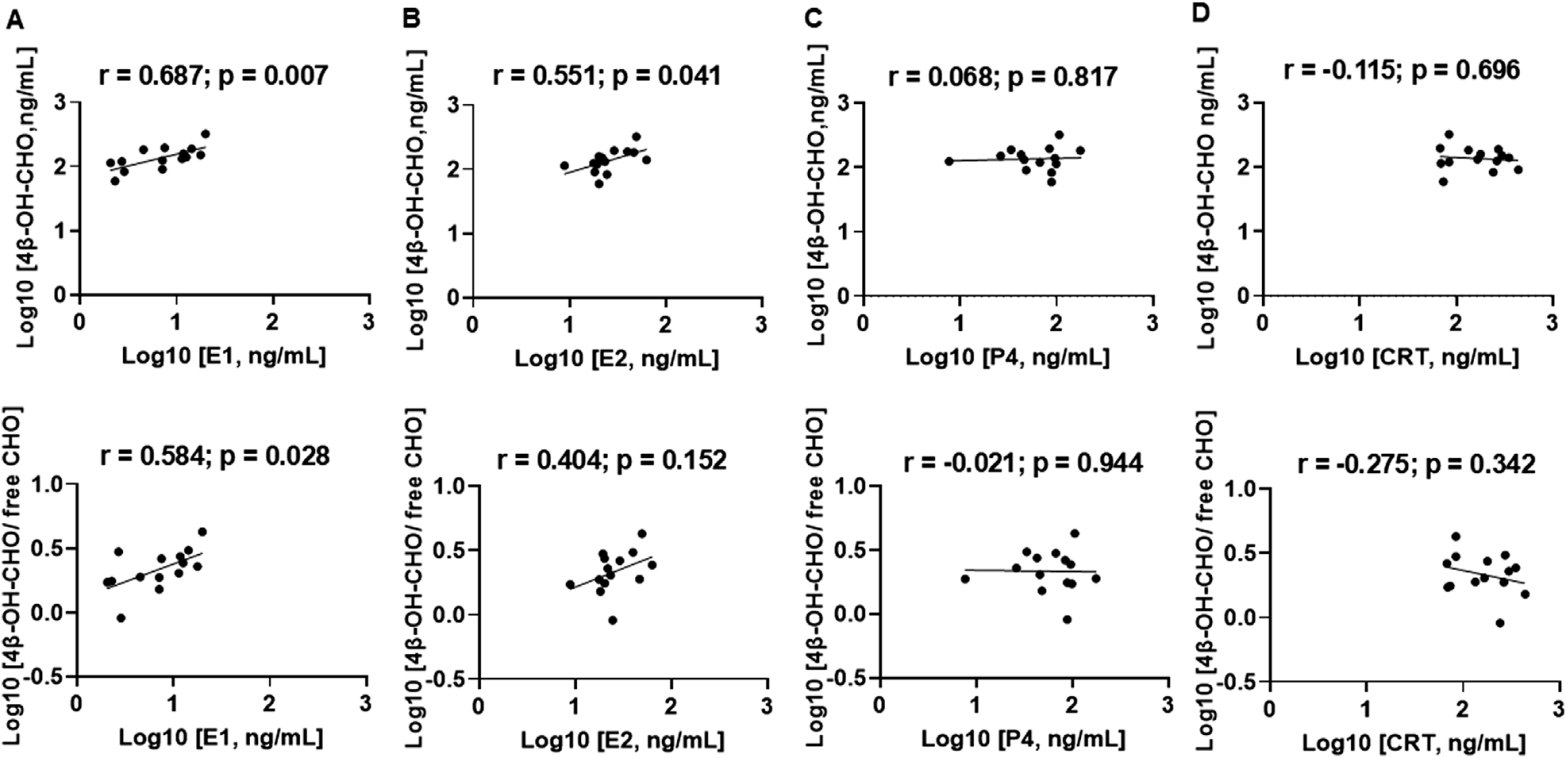
Correlation between steroidal pregnancy related hormones and 4β-hydroxycholesterol biomarkers of CYP3A activity in maternal circulation. The analysis included plasma data from healthy pregnant volunteers and preeclampsia patients (n = 14). Correlations were performed between plasma concentrations of hormones **(A)** estrone (E1), **(B)** estradiol (E2), **(C)** progesterone (P4), and **(D)** cortisol (CRT) and 4β-OH-CHO (upper panel) and the ratio of 4β-OH-CHO to free CHO (lower panel). The Pearson correlation analysis was carried out on log-transformed data, and the correlation coefficient (*r*) and *p* values are presented.

To investigate the association between inter-individual variation in steroidal PRH exposure and CYP3A activity during pregnancy in humans, correlations between circulating concentrations of E1, E2, P4, and CRT and 4β-OH-CHO biomarkers of CYP3A activity in maternal plasma (healthy pregnant and preeclampsia groups combined) were evaluated (Figure 3). Plasma E1 (Figure 3A, *r = 0.687; p = 0.007*) and E2 (Figure 3B, *r = 0.551; p = 0.041*) concentrations were significantly and positively correlated with the plasma 4β-OH-CHO concentrations in pregnant individuals. Plasma E1 concentrations and the 4β-OH-CHO:CHO ratio were also significantly correlated (Figure 3A, *r = 0.584; p = 0.028*); plasma E2 concentrations exhibited a positive, but not statistically significant, correlation with the 4β-OH-CHO:CHO ratio (Figure 3B, *r = 0.404; p = 0.152*). Unlike the estrogens, P4 (Figure 3C) and CRT (Figure 3D) plasma concentrations did not exhibit a correlation with either 4β-OH-CHO concentrations or the 4β-OH-CHO:CHO ratio in maternal plasma. The PRH correlations were also evaluated exclusively within the healthy pregnant group (Supplementary Table S4), which were overall consistent with results from the analysis that combined the healthy pregnant and preeclampsia groups.

### PRHs induce CYP protein concentrations in human primary hepatocytes in an isoform-specific manner

**FIGURE 4:**
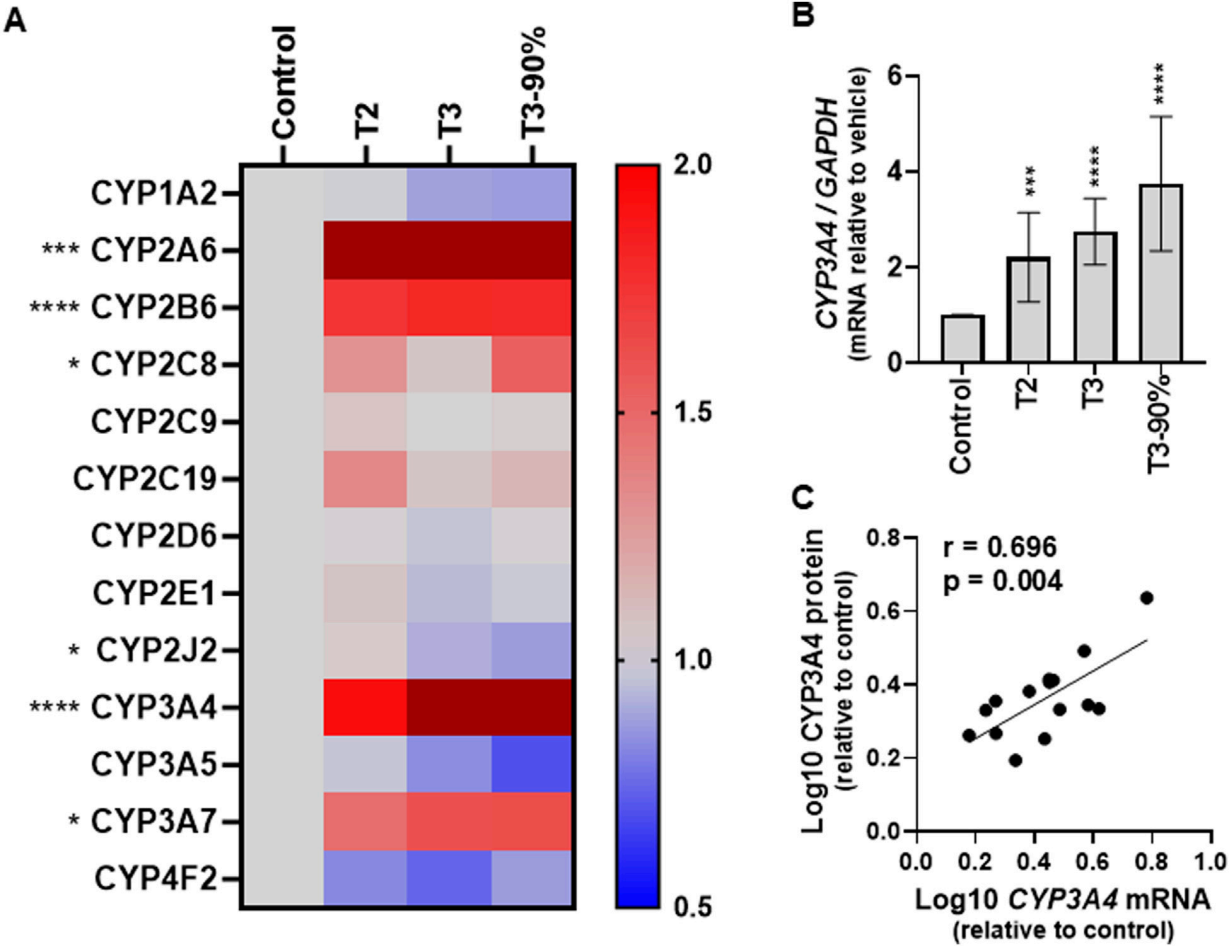
Impact of pregnancy-related hormones (PRHs) on the absolute protein concentrations of 13 major cytochrome P450 enzymes, and mRNA levels of CYP3A4, in sandwich-cultured human hepatocytes (SCHH). SCHH from five qualified female donors (Supplementary Table S1) were exposed to vehicle and pregnancy related hormone (PRH) cocktails (targeting average trimester 2 (T2), average trimester 3 (T3), and upper range of T3 (T3-90%) concentrations). In each hepatocyte donor, the fold-difference (fd) for each CYP protein or *CYP3A4* mRNA (relative to vehicle control) was calculated for each PRH concentration (n = 3–4 biological replicates). The average fd within in each hepatocyte donor was carried forward as a single data point into an analysis of the net effect of each PRH cocktail across donors. **(A)** The heat map presents the mean net increase (red), no change (gray), or decrease (blue) in absolute protein concentration for each of the 13 CYP isoforms across the five donors relative to vehicle control. Color index: Deep red (fd > 2) Red (fd > 1), Gray, (fd = 1), Blue (fd < 1). ANOVA *p < 0.05, **p < 0.01, ***p < 0.001, ****p < 0.0001 comparisons across the vehicle control and 3 PRH treatment groups for each CYP (fold-differences and inter-group comparisons are presented in Supplementary Table S5). **(B)** The bar graphs represent the mean (± standard error of mean) fd in *CYP3A4* mRNA levels following exposure to PRH cocktails across the five donors relative to vehicle control. ***p < 0.001, ****p < 0.0001 *versus* control. **(C)** Correlation between CYP3A4 protein concentrations and *CYP3A4* mRNA levels (log-transformed). The Pearson correlation coefficient (*r*) and *p* value are presented.

The absolute concentration of 13 CYP proteins were quantified in SCHH exposed to PRH cocktails that target the average trimester 2 (T2), average trimester 3 (T3), and the upper range of trimester 3 (T3-90%) plasma concentration of each PRH in maternal circulation. A heatmap presenting the average PRH impact of each PRH cocktail, relative to vehicle control, on CYP isoform protein concentration across donors revealed isoform-specific and concentration-dependent effects (Figure 4A). The corresponding average fold-difference of each CYP protein in each PRH treatment group is summarized in Supplementary Table S5.

Overall, 6 of 13 (46%) CYP proteins exhibited a significant PRH-evoked net change in protein concentration relative to vehicle control (*ANOVA p < 0.05*) across the 5 hepatocyte donors. The PRH cocktail increased CYP2A6 (*p < 0.001*), CYP2B6 (*p < 0.001*), CYP2C8 (*p = 0.015*), CYP3A4 (*p < 0.001*), and CYP3A7 (*p = 0.041*) and decreased CYP2J2 (*p = 0.022*) protein concentrations in SCHH (Supplementary Table S5). Relative to control, the magnitude of the PRH cocktail induction effect was greatest with CYP3A4 (2.35 ± 0.48-fold at the T3 concentration), followed by CYP2A6 (2.02 ± 0.46-fold), CYP2B6 (1.81 ± 0.21-fold), and CYP3A7 (1.61 ± 0.50-fold). The reduction in CYP2J2 was small (0.87 ± 0.07-fold) and CYP1A2, CYP2C9, CYP2C19, CYP2D6, CYP2E1, CYP3A5, and CYP4F2 protein concentrations were not altered by PRHs.

Consistent with the observed PRH-mediated increases in CYP3A4 protein concentration, PRH exposure increased *CYP3A4* mRNA levels in a concentration-dependent manner (Figure 4B). PRH-induced changes CYP3A4 protein concentration and *CYP3A4* mRNA levels were positively correlated (Figure 4C, *r = 0.696; p = 0.004*).

### Estrogens (E1 and E2) induce CYP3A4 protein expression in human primary hepatocytes

**FIGURE 5:**
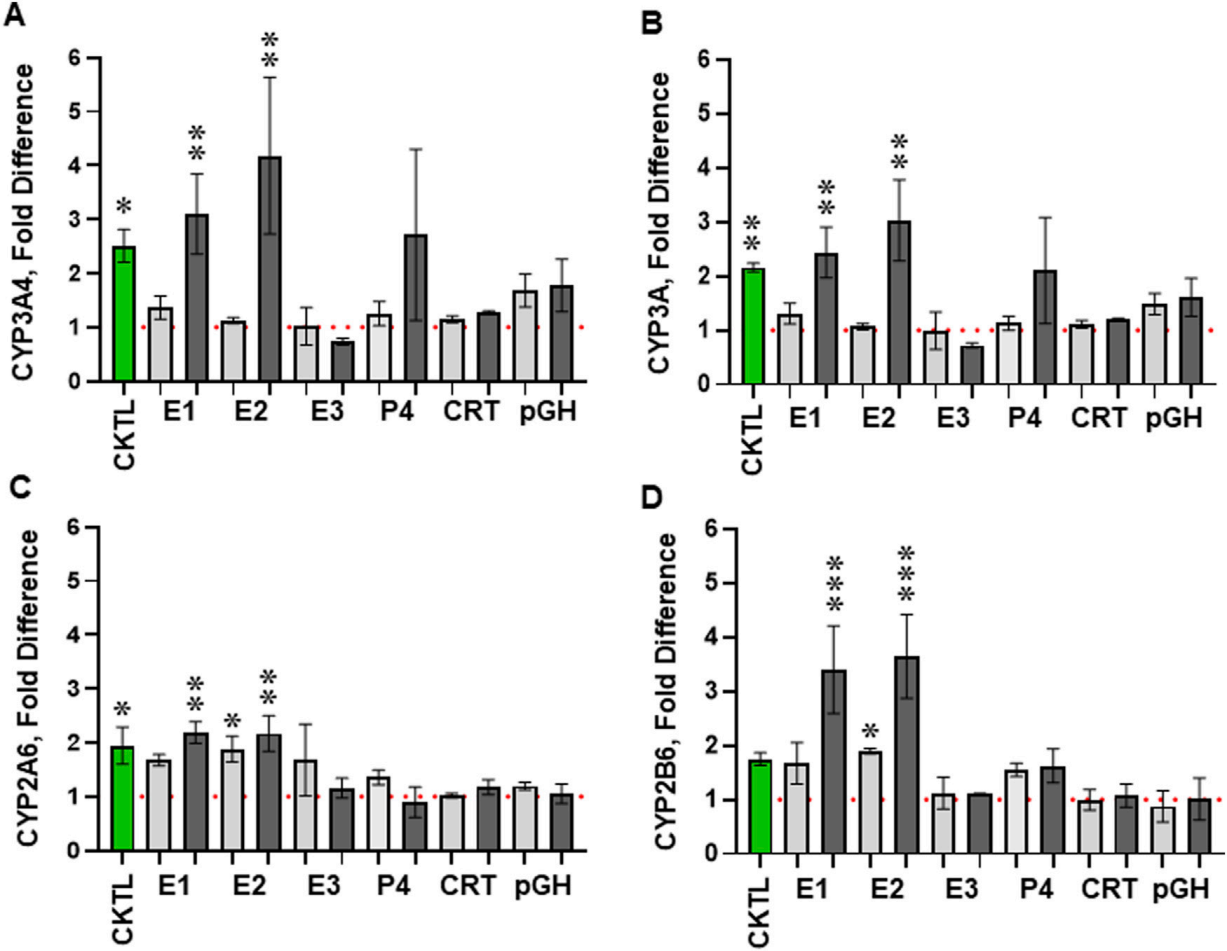
Impact of individual pregnancy related hormones (PRHs) on CYP3A4, total CYP3A, CYP2A6, and CYP2B6 absolute protein concentrations in sandwich-cultured human hepatocytes (SCHH). SCHH from three qualified donors (Hu8373, Hu8375, and Hu1970) were exposed to vehicle control, PRH cocktail (CKTL, green bar) that targets average trimester 3 (T3) PRH concentrations, or individual PRHs (E1, E2, E3, P4, CRT, or pGH) that target average T3 (light gray bars) or supraphysiologic 10x-T3 (dark gray bars) PRH concentrations (n = 3–4 biological replicates in each donor). The bar graphs depict the mean (± standard error of mean) fold-difference relative to vehicle control in **(A)** CYP3A4, **(B)** total CYP3A (CYP3A4+CYP3A5+CYP3A7), **(C)** CYP2A6, **(D)** CYP2B6 absolute protein concentration across the hepatocyte donors (n = 3) in response to the T3 PRH CKTL or individual PRHs at T3 and 10x-T3 concentrations. The dashed line depicts the vehicle control. *p < 0.05, **p < 0.01, ***p < 0.001 *versus* vehicle control.

To evaluate the concentration-dependent effect of individual PRHs on CYP3A4, total CYP3A, CYP2A6, and CYP2B6 protein concentrations, SCHH from 3 hepatocyte donors were exposed to the individual PRHs E1, E2, E3, P4, CRT, and pGH at average T3 and supraphysiological (10x-T3) PRH concentrations. The T3 PRH cocktail (used in Figure 4), which is a combination of each individual hormone at the T3 concentration (CKTL), was included in this experiment for reference; the T3 PRH CKTL increased CYP3A4, total CYP3A, CYP2A6, and CYP2B6 protein concentrations (Figure 5).

In the individual PRH treatments, exposure to E1 and E2 each increased CYP3A4 (Figure 5A) and total CYP3A (Figure 5B) protein in a concentration-dependent manner. Relative to vehicle control, E1 treatment at average T3 and 10x-T3 concentrations increased CYP3A4 protein concentrations by 1.37 ± 0.37-fold and 3.11 ± 1.29-fold and total CYP3A protein concentrations by 1.31 ± 0.33-fold and 2.44 ± 0.80-fold, respectively. Similarly, E2 treatment increased CYP3A4 by 1.12 ± 0.10-fold and 4.18 ± 2.52-fold and total CYP3A by 1.07 ± 0.10-fold and 3.30 ± 1.3-fold, respectively. Consistent with the observed effects on CYP3A4, E1 and E2 each also increased CYP2A6 (Figure 5C), and CYP2B6 (Figure 5D) protein concentrations. In contrast, E3, P4, CRT, and PGH each did not significantly alter CYP3A4, CYP2A6, or CYP2B6 protein concentrations in SCHH.

## Discussion

The key factors that contribute to increased CYP3A activity and clearance of CYP3A substrates during human pregnancy are not well understood. Although PRHs increase CYP3A4 expression and activity in human hepatocytes *in vitro* (Choi et al., 2013; Papageorgiou et al., 2013; Khatri et al., 2021; Fashe et al., 2023), the association between PRHs and increased CYP3A activity *in vivo* remains unclear. Here, we studied the association between maternal plasma concentrations of key steroidal PRHs (E1, E2, P4, and CRT) and endogenous 4β-OH-CHO biomarkers of CYP3A activity (4β-OH-CHO concentration, 4β-OH-CHO:CHO ratio) across a spectrum of pregnancy states (nonpregnant, healthy pregnant, pregnant diagnosed with preeclampsia) in humans, and the impact of PRHs on CYP3A4 protein expression in SCHH. The major findings were: (1) plasma concentrations of E1 and E2, but not P4 or CRT, positively correlated with plasma 4β-OH-CHO biomarkers of CYP3A activity, (2) PRHs evoked a greater fold-increase in CYP3A4 absolute protein concentrations compared to other CYP isoforms in SCHH, and (3) consistent with the human plasma sample associations, exogenous exposure to E1 and E2 increased CYP3A4 and total CYP3A protein concentrations in SCHH. Collectively, these data suggest that gestation-associated increases in estrogens contribute, at least in part, to enhanced hepatic CYP3A expression and activity during human pregnancy.

The markedly higher plasma concentrations of steroidal PRHs (E1, E2, P4, and CRT) in pregnant individuals compared to nonpregnant controls in our study population were expected and consistent with previous reports (Newbern and Freemark, 2011). Prior studies have reported lower circulating concentrations of estrogens in severe, but not mild, preeclampsia relative to normotensive pregnancies (Jobe et al., 2013). In our study, E1 concentrations were lower in preeclampsia compared to healthy pregnancy, while E2 concentrations were comparable between groups. Consistent with reports that preeclampsia is associated with approximately 2-fold higher circulating P4 concentrations (Moon et al., 2014) and lower circulating CRT concentrations (Ho et al., 2007; Kosicka et al., 2018), we observed 2.4-fold higher P4 and 0.3-fold lower CRT plasma concentrations in pregnant patients diagnosed with preeclampsia relative to healthy pregnancy. Although our observations with E1, P4, and CRT are consistent with prior studies, it is important to acknowledge that there was a difference in the median gestational age between the healthy pregnancy (27.1 weeks) and preeclampsia (31.4 weeks) groups in our study that could have influenced the hormone comparisons and contributed to the lack of an observed difference in E2 concentrations across groups.

Plasma 4β-OH-CHO concentrations and the 4β-OH-CHO:CHO ratio are routinely used as endogenous biomarkers of CYP3A activity in different disease and physiological states including pregnancy (Kim et al., 2018; Gravel et al., 2019; Mlugu et al., 2022; Lee et al., 2024). Evaluation of 4β-OH-CHO:CHO, and not 4β-OH-CHO concentrations alone, is important in conditions where CHO levels vary among the study population (Diczfalusy et al., 2011). In our study population, we observed pregnancy associated increases in both 4β-OH-CHO and the 4β-OH-CHO:CHO ratio, despite also observing higher CHO, which was consistent with prior observations that pregnancy increases these circulating CYP3A biomarkers by approximately 1.5-2 fold during gestation (Kim et al., 2018; Mlugu et al., 2022). In adult humans, total CYP3A activity reflects CYP3A4 and CYP3A5 function (Kuehl et al., 2001), and changes in activity of each isoform can introduce interindividual differences in CYP3A activity. Transcriptional induction and inhibition are major contributors to variation in CYP3A4 activity, while germline genetic polymorphisms are the major source of variation in CYP3A5 expression and activity (Roy et al., 2005; Hakkola et al., 2020). CYP3A5 expression status did not predict increases in CYP3A activity during pregnancy (Mlugu et al., 2022), suggesting that increased CYP3A4 activity is the primary contributor to increased plasma 4β-OH-CHO and 4β-OH-CHO:CHO during gestation. Although the mechanisms underlying increased CYP3A4 activity during pregnancy *in vivo* remain unclear, it has been hypothesized that these effects are mediated by increased secretion of PRHs (Jeong, 2010; Isoherranen and Thummel, 2013). Our SCHH experiments demonstrate that PRHs induce *CYP3A4* mRNA and CYP3A4 protein, which strongly correlate with CYP3A activity (Fashe et al., 2023), to a greater degree than other CYP isoforms.

In our study population of healthy pregnant volunteers and pregnant patients diagnosed with preeclampsia, plasma concentrations of estrogens (E1 and E2), but not P4 or CRT, positively correlated with 4β-OH-CHO biomarkers of CYP3A activity. Consistent with this observation in human plasma samples, exogenous administration of E1 and E2 increased CYP3A4 (and total CYP3A) protein concentration in SCHH. It is important to note that the individual E1 and E2 effects on CYP3A4 protein expression were only apparent at the supraphysiological 10x-T3 concentration in our *in vitro* model. This indicates that while E1 and E2 individually contribute to PRH induction of hepatic CYP3A4 in a concentration-dependent manner, the estrogens are not solely responsible and the combinatorial effects of multiple hormones (that include E1 and E2) likely mediate transcriptional induction of hepatic CYP3A4 expression during pregnancy. The current study was limited to the major steroidal PRHs (E1, E2, P4, CRT) due to the well-established role of steroid hormones on nuclear receptor-mediated regulation of CYP3A4. However, we acknowledge that pregnancy-mediated changes in hepatic CYP3A expression and activity *in vivo* could result from alterations in other PRHs (including non-steroidal hormones such as human chorionic gonadotropin [hCG], human placental lactogen [hPL], and prolactin). Future studies are needed to discern these effects and interactions *in vitro* and *in vivo*.

The mechanisms of estrogen-mediated increases in CYP3A4 have not been established but could be mediated by (1) increased transcriptional induction of *CYP3A4*, (2) posttranslational modifications, and/or (3) enhanced enzyme activity through heterotropic cooperative effects. Cooperative stimulation and posttranslational modification of CYP3A4, and the *in vivo* relevance of these processes, are not well understood (Tang and Stearns, 2001; Wang et al., 2009). It is well-established that basal and induced expression of CYP3A4 are regulated by multiple transcription factors such as hepatocyte nuclear factor-4alpha (HNF4α), ERα, PXR, CAR, glucocorticoid receptor (GR), growth hormone receptor (GHR), and retinoid X receptor *α* (RXR*α*) (Tirona et al., 2003; Waxman and O'Connor, 2006; Istrate et al., 2010; Qin and Wang, 2019; Wang et al., 2019). However, the molecular mechanisms underlying nuclear receptor-mediated regulation of increased CYP3A4 expression during pregnancy remain unclear.

Consistent with prior experiments in similar models (Choi et al., 2013; Khatri et al., 2021), our *in vitro* data in SCHH indicate that estrogens can induce CYP3A4, CYP2B6, and CYP2A6 expression in a concentration-dependent manner. It is well-established that estrogens elicit their actions by binding to and activating their natural receptor ERα. Activated ERα translocates to the nucleus, forms a homodimer, and binds to its estrogen response element (ERE) on established target genes such as *CYP2A6* and *CYP2B6*. ERα directly binds its EREs on the regulatory region of *CYP2A6* (−2,436/−2,424) (Higashi et al., 2007) and *CYP2B6* (−1,669/−1,657) (Lo et al., 2010) and each gene is induced following exposure to estrogens in human liver cells. Consistent with these effects, and similar to CYP3A4, both E1 and E2 also increased CYP2A6 and CYP2B6 protein concentrations in our SCHH experiments. Although the direct binding of ERα to the *CYP3A4* promoter *via* presence of a putative ERE has not been established, ERα can serve as a key regulator of *CYP3A4* transcription by interacting with other transcription factors, such as PXR and CAR (Wang et al., 2019). In human pregnancy, however, it remains unclear whether an estrogen-evoked increase in hepatic CYP3A4 expression and activity is mediated by activation of xenosensors PXR and CAR, natural estrogen receptor ERα, or both. In diabetic mouse liver, CAR is constitutively activated and recruits ERα/ERα homodimer to the *Sult1e1* promoter to form the CAR/RXR-ERα/ERα tetramer and induce *Sult1e1* (Negishi et al., 2020; Yi et al., 2020). Although this suggests that estrogens could similarly activate both CAR and ERα to cooperatively activate transcriptional induction of *CYP3A4* during gestation, future studies elucidating these mechanisms are needed.

Induction of *CYP3A4* by glucocorticoids is well-established. A previous study reported that CRT induced *CYP3A4* mRNA levels in HepaRG cells occurs predominantly through GR facilitated upregulation of PXR (Sachar et al., 2019). In our study, however, we did not observe evidence of a correlation between concentrations of CRT and 4β-OH-CHO biomarkers in plasma collected from pregnant individuals. Similarly, although a prior study reported P4 induction of CYP3A4 expression and activity in human hepatocytes (Choi et al., 2013), P4 did not positively correlate with 4β-OH-CHO plasma biomarkers during gestation. An important limitation of our study was the limited sample size in the non-pregnant, healthy pregnancy, and preeclampsia groups and the lack of pregnant participants also spanning each trimester and post-partum, which limited the statistical power and precision of our correlation analysis. Thus, we cannot confidently conclude that no relationship between CYP3A activity and CRT and P4 exists. Due to the limited sample size, we also cannot confidently discern whether the magnitude of the observed associations differ across hormones (e.g., E1 *versus* E2) or whether the observed PRH-CYP3A activity associations are influenced by preeclampsia status and significantly differ across the healthy pregnancy and preeclampsia groups. Moreover, we did not quantify other endogenous factors (such as inflammatory cytokines) or concomitant medication use (such as nifedipine or labetalol) at the time of blood sampling that could influence CYP3A activity and impact 4β-OH-CHO associations with PRHs in patients with preeclampsia. We also did not measure plasma concentrations of E3 and pGH, or other non-steroidal hormones (such as hCG, hPL, or prolactin), due to lack of qualified analytical methods and limited plasma samples from pregnant individuals. Although E3 and pGH did not induce CYP3A4 expression in SCHH, it remains unknown if these or other hormones are associated with altered CYP3A activity during pregnancy in humans. It is important to consider these limitations when interpreting the correlation analysis in the current study, which should be considered hypothesis-generating until validated in a larger independent population. Additional studies that investigate associations of steroidal and non-steroidal PRHs, inflammatory cytokines, and other factors with CYP3A activity in larger and more diverse pregnancy populations are warranted.

## Conclusion

In summary, this study investigated the relationship between key steroidal PRHs and CYP3A-mediated metabolism in human pregnancy and in human hepatocytes *in vitro*. We observed that higher circulating maternal plasma concentrations of estrogens (E1 and E2) were associated with higher plasma 4β-OH-CHO biomarkers of CYP3A activity in human pregnancy. Experiments in female SCHH demonstrated that exogenous exposure to E1 and E2 increased CYP3A4 protein concentrations in a concentration-dependent manner; similar increases in CYP2A6 and CYP2B6 expression, which are well-known to be regulated by estrogens, were observed. Together, these results suggest that increased concentrations of E1 and E2 contribute, at least in part, to increases in hepatic CYP3A expression and activity observed during human pregnancy.

## Data Availability

The raw data supporting the conclusions of this article will be made available by the authors, without undue reservation.

## Abbreviations

4*β*-OH-CHO, 4*β*-hydroxycholesterol; CHO, cholesterol; 4*β*-OH-CHO:CHO, ratio of 4*β*-OH-CHO to CHO; CAR, constitutive androstane receptor; CRT, cortisol; CYP, cytochrome P450; E1, estrone; E2, estradiol; ER, estrogen receptor; ERE, estrogen response element; GR, glucocorticoid receptor; GHR, growth hormone receptor; P4, progesterone; pGH, placental growth hormone; PRH, pregnancy related hormones; PXR, pregnane X receptor; QTAP, quantitative targeted absolute proteomics; RXR*α*, retinoid X receptor *α*.
