# Supplementary material for: Estrogen associations with human pregnancy related increases in cytochrome P450 3A activity
Source: Front Pharmacol. 2025 Nov 26;16:1702419. doi: 10.3389/fphar.2025.1702419 (PMC12689976; doi:10.3389/fphar.2025.1702419)
Supplement: Supplementary file 1 [file DataSheet1.pdf]

Supplemental Material

Estrogen Associations with Human Pregnancy Related Increases in Cytochrome P450 3A Activity

Muluneh M. Fashe,[1] Jonghwa Lee,[1] Joseph T. Grieco,[1] John K. Fallon,[2] Ian R. Mulrenin,[1] Megan N. Gower[1], Klarissa D. Jackson,[1] Kim A. Boggess[3], Craig R. Lee[1]*

[1]Division of Pharmacotherapy and Experimental Therapeutics, UNC Eshelman School of Pharmacy, University of North Carolina at Chapel Hill, Chapel Hill, NC, United States

[2]Division of Pharmacoengineering and Molecular Pharmaceutics and Center for Nanotechnology in Drug Delivery, UNC Eshelman School of Pharmacy, University of North Carolina at Chapel Hill, Chapel Hill, NC, United States

[3]Department of Obstetrics & Gynecology, School of Medicine, University of North Carolina at Chapel Hill, Chapel Hill, North Carolina, United States

***Correspondence:**

Craig R. Lee, Pharm.D., Ph.D.

craig_lee@unc.edu

Supplementary Tables: 5

SUPPLEMENTARY INFORMATION

Supplementary Table S1. Primary hepatocyte donor characteristics (all donors are female).

Donors	Age (yr.)	Race	Vendor	Transporter qualified	Induction qualified	Drug history	Cause of Death
**Hu8339**	31	African American	Life Technologies	Yes	Yes	Marijuana	Asphyxiation
**Hu8373**	26	White	Life Technologies	No	Yes	Cocaine, cannabis	Asphyxiation
**Hu8375**	19	White	Life Technologies	Yes	Yes	Cannabis	Asphyxiation
**YNM**	48	White	BioIVT	Yes	Yes	Not reported	Anoxia
**Hu1970**	34	White	Life Technologies	Yes	Yes	Not reported	Not reported

Supplementary Table S2. Tryptic peptides, their sequences, and SIL peptide MRMs used to report the absolute protein concentration of major cytochrome P450s in sandwich-cultured human hepatocytes.

Enzyme	Peptide Sequence & position	MRM1	MRM2
**CYP1A2**	Y$_{244}$LPNPALQ**R**$_{252}$	541.31/403.23(y7)	541.31/594.36(y5)
**CYP2A6**	G$_{162}$TGGANIDPTFFLS**R**$_{176}$	781.89/877.49(y7)	781.89/992.51(y8)
**CYP2B6**	G$_{379}$YIIP**K**$_{384}$	349.72/478.35(y4)	349.72/365.26(y3)
**CYP2C8**	N$_{466}$LNTTAVT**K**$_{474}$	485.28/742.42(y7)	485.28/628.38(y6)
**CYP2C9**	G$_{98}$IFPLAE**R**$_{105}$	456.76/595.34(y5)	456.76/298.18(y5)
**CYP2C19**	G$_{98}$HFPLAE**R**$_{105}$	312.84/385.21(y3)	312.84/439.21(b4)
**CYP2D6**	S$_{116}$QGVFLA**R**$_{123}$	444.25/672.41(y6)	444.25/516.63(y4)
**CYP2E1**	F$_{360}$ITLVPSNLPHEAT**R**$_{374}$	568.98/566.29(y10)	568.98/720.37(y6)
**CYP2J2**	D$_{276}$FIDAYL**K**$_{283}$	496.76/730.43(y6)	496.76/617.34(y5)
**CYP3A4**	L$_{477}$SLGGLLQPEKPVVL**K**$_{492}$	567.03/693.43(y13)	567.03/664.92(y12)
**CYP3A5**	D$_{244}$TINFLS**K**$_{251}$	473.26/729.44(y6)	473.3/217.1(b2)
**CYP3A7**	E$_{334}$IDTVLPN**K**$_{342}$	518.79/366.22(y3)	518.79/794.45(y7)
**CYP4F2**	S$_{109}$VINASAAIAP**K**$_{120}$	575.34/850.49(y9)	575.34/665.41(y7)

SIL peptides were obtained from JPT (Berlin, Germany)

Supplementary Table S3. Characteristics of study participants.

	**Healthy Nonpregnant** *n=4*	**Healthy Pregnant** *n=6*	**Pregnant with Preeclampsia** *n=8*
Age (years)	24.5 (23.3, 25.8)	33.5 (32.5, 34.0)	29.0 (20.3, 34.5)
Gestational Age (weeks)	N/A	27.1 (25.6, 27.9)	31.4 (29.8, 32.3)
Body Mass Index (kg/m^2)	25.1 (20.9, 30.5)	26.4 (23.6, 29.4)	31.2 (25.2, 39.9)
Race/Ethnicity			
White	1 (25%)	5 (83.3%)	3 (37.5%)
Asian	1 (25%)	0 (0.0%)	0 (0.0%)
Black	0 (0.0%)	0 (0.0%)	3 (37.5%)
Hispanic/Latino	2 (50%)	1 (16.7%)	2 (25%)

N/A, data not applicable

Supplementary Table S4. Correlation between circulating steroidal pregnancy related hormones and 4β-hydroxycholesterol biomarkers of CYP3A activity in healthy pregnant volunteers.

**PRH**	**4β-OH-CHO**		**4β-OH-CHO : CHO ratio**	
	**r**	**p-value**	**r**	**p-value**
E1	r = 0.784	p = 0.065	r = 0.849	p = 0.032
E2	r = 0.730	p = 0.099	r = 0.614	p = 0.195
P4	r = -0.003	p = 0.995	r = 0.275	p = 0.598
CRT	r = -0.494	p = 0.319	r = -0.706	p = 0.117

The analysis included plasma data from healthy pregnant volunteers only (n=6). Correlations were performed between plasma concentrations of hormones estrone (E1), estradiol (E2), progesterone (P4), and cortisol (CRT) and both 4β-OH-CHO and the ratio of 4β-OH-CHO to free CHO. The Pearson correlation analysis was carried out on log-transformed data, and the correlation coefficient (r) and p values are presented.

Supplementary Table S5. Fold-differences of 13 major CYP protein concentrations in sandwich-cultured human hepatocytes exposed to pregnancy related hormone cocktails.

	*ANOVA P-value**	**T2**		**T3**		**T3-90%**	
		Mean ± SD	*P^*	*Mean ± SD*	*P^*	*Mean ± SD*	*P^*
**CYP1A2**	*0.506*	0.99 ± 0.31		0.88 ± 0.16		0.87 ± 0.11	
**CYP2A6**	*0.0002*	2.14 ± 0.75	*<0.001*	2.02 ± 0.46	*<0.001*	2.03 ± 0.48	*<0.001*
**CYP2B6[#]**	*<0.0001*	1.74 ± 0.17	*<0.001*	1.81 ± 0.21	*<0.001*	1.80 ± 0.38	*<0.001*
**CYP2C8**	*0.015*	1.29 ± 0.15	*0.049*	1.06 ± 0.19	*0.725*	1.54 ± 0.49	*0.005*
**CYP2C9**	*0.605*	1.07 ± 0.16		1.00 ± 0.06		1.03 ± 0.08	
**CYP2C19**	*0.377*	1.34 ± 0.47		1.06 ± 0.19		1.13 ± 0.30	
**CYP2D6**	*0.961*	1.01 ± 0.16		0.96 ± 0.04		1.01 ± 0.19	
**CYP2E1**	*0.390*	1.07 ± 0.21		0.94 ± 0.08		0.98 ± 0.07	
**CYP2J2**	*0.022*	1.03 ± 0.12	*0.652*	0.91 ± 0.08	*0.099*	0.87 ± 0.07	*0.018*
**CYP3A4**	*<0.0001*	1.92 ± 0.26	*<0.001*	2.35 ± 0.48	*<0.001*	2.85 ± 0.85	*<0.001*
**CYP3A5**	*0.075*	0.97 ± 0.24		0.84 ± 0.14		0.69 ± 0.24	
**CYP3A7**	*0.041*	1.48 ± 0.65	*0.062*	1.61 ± 0.50	*0.016*	1.63 ± 0.34	*0.011*
**CYP4F2**	*0.147*	0.83 ± 0.16		0.74 ± 0.21		0.87 ± 0.12	

The fold-differences were estimated relative to the vehicle control group within each hepatocyte donor and then averaged within in each treatment group across the 5 donors.

**The ANOVA p-value for the comparison across the vehicle control and 3 PRH treatment groups (n=5 donors) is reported. ^For proteins with an overall ANOVA p < 0.05, the post-hoc Fisher's LSD p-value for the comparison or each PRH group versus vehicle control is reported. [#]CYP2B6 absolute protein concentration was detectable but below the lower limit of quantitation (0.1 pmol/mg) in hepatocyte donor Hu8339 (in each treatment group); therefore, the reported fold-difference comparison for CYP2B6 was across 4 hepatocyte donors.*
